# Dual Host-Virus Arms Races Shape an Essential Housekeeping Protein

**DOI:** 10.1371/journal.pbio.1001571

**Published:** 2013-05-28

**Authors:** Ann Demogines, Jonathan Abraham, Hyeryun Choe, Michael Farzan, Sara L. Sawyer

**Affiliations:** 1 Department of Molecular Genetics and Microbiology, Institute for Cellular and Molecular Biology, University of Texas at Austin, Austin, Texas, United States of America; 2 Department of Medicine, Children's Hospital, Harvard Medical School, Boston, Massachusetts, United States of America; 3 Department of Microbiology and Molecular Genetics, Harvard Medical School, Boston, Massachusetts, United States of America; University of Wisconsin-Madison, United States of America

## Abstract

Transferrin Receptor (TfR1) is the cell-surface receptor that regulates iron uptake into cells, a process that is fundamental to life. However, TfR1 also facilitates the cellular entry of multiple mammalian viruses. We use evolutionary and functional analyses of TfR1 in the rodent clade, where two families of viruses bind this receptor, to mechanistically dissect how essential housekeeping genes like *TFR1* successfully balance the opposing selective pressures exerted by host and virus. We find that while the sequence of rodent TfR1 is generally conserved, a small set of TfR1 residue positions has evolved rapidly over the speciation of rodents. Remarkably, all of these residues correspond to the two virus binding surfaces of TfR1. We show that naturally occurring mutations at these positions block virus entry while simultaneously preserving iron-uptake functionalities, both in rodent and human TfR1. Thus, by constantly replacing the amino acids encoded at just a few residue positions, *TFR1* divorces adaptation to ever-changing viruses from preservation of key cellular functions. These dynamics have driven genetic divergence at the *TFR1* locus that now enforces species-specific barriers to virus transmission, limiting both the cross-species and zoonotic transmission of these viruses.

## Abstract 2

Relentless selective pressures exerted by viruses trigger arms race dynamics that shape the evolution of even critical host genes like those involved in iron homeostasis.

## Author Summary

Genetic differences between mammalian species dictate the patterns of viral infection observed in nature. They also define how viruses must evolve in order to infect new mammalian hosts, giving rise to new and sometimes pandemic diseases. Because viruses must enter cells before they can replicate, new diseases often emerge when existing viruses evolve the ability to bind to the cell-surface receptor of a new species. At the same time, host cell receptors also evolve to counteract virus attacks. This back-and-forth evolution between virus and host can lead to an arms race that shapes the sequences of the proteins involved. In wild rodent populations, the retrovirus MMTV and New World arenaviruses both exploit Transferrin Receptor 1 (TfR1) to enter the cells of their hosts. Here we show that the physical interactions between these viruses and TfR1 have triggered evolutionary arms race dynamics that have directly modified the sequence of TfR1 and at least one of the viruses involved. Computational evolutionary analysis allowed us to identify specific residues in TfR1 that define patterns of viral infection in nature. The approach presented here can theoretically be applied to the study of any virus, through analysis of host genes known to be key to controlling viral infection. As such, this approach can expand our understanding of how viruses emerge from wildlife reservoirs, and how they drive the evolution of host genes.

## Introduction

Transferrin receptor (TfR1) is the cell-surface receptor for iron-loaded transferrin circulating in the blood [1]. TfR1-transferrin complexes are internalized via clathrin-mediated endocytosis and iron is released in acidic endosomes. Besides transferrin, the other major binding partner of TfR1 is the hereditary hemochromatosis protein (HFE), which negatively regulates iron uptake. In addition to these host-beneficial interactions, three different families of viruses are known to interact with TfR1 to trigger their own cellular entry. TfR1 likely constitutes an attractive target for viruses because it is both ubiquitous and specifically up-regulated in rapidly dividing cells [1]. Because of the tremendous investment that has been made in understanding both TfR1 and the viruses that exploit it, there are rich structural and functional data available. For instance, co-crystal structures have been solved of human TfR1 in complex with both of its cellular iron-transport binding partners [2]–[4] and with the surface glycoprotein of a zoonotic rodent arenavirus, Machupo virus, which uses TfR1 for entry [5]. For this reason, TfR1 provides an ideal opportunity to investigate how cellular housekeeping proteins evolve to combat viruses that are exploiting them while simultaneously preserving critical cellular functions.

**Figure 1:**
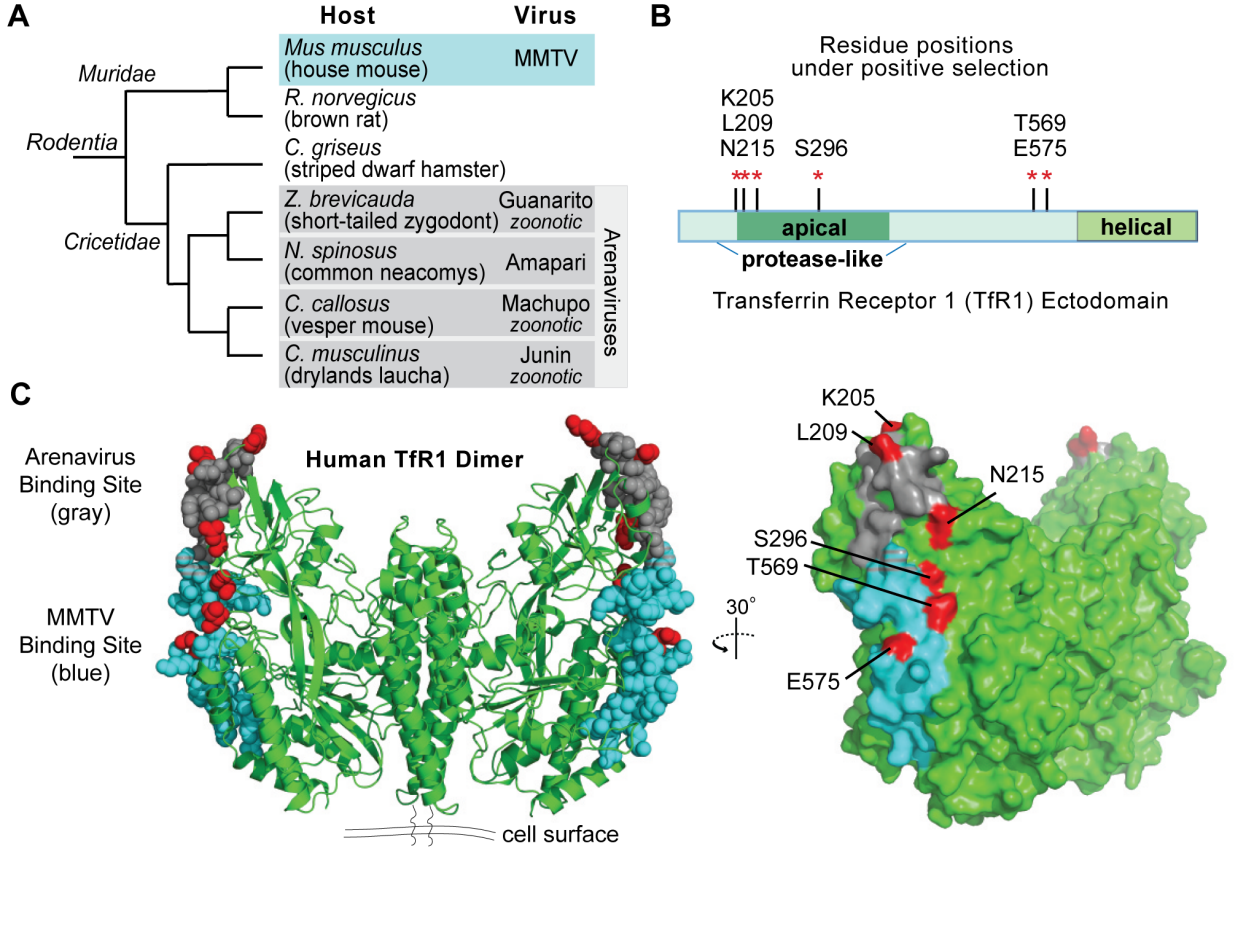
*TFR1* evolution in rodents has been shaped by two separate host-virus arms races. (A) A cladogram illustrates the evolutionary relationship of the rodent species analyzed. These species fall into two major families: *Muridae* and *Cricetidae*. The retrovirus (MMTV) and arenaviruses known to be harbored by these rodents in nature are also indicated. Three of the rodent arenaviruses (Guanarito, Machupo, and Junin) are zoonotically transmitted to humans. (B) Red stars represent the six rapidly evolving codon positions identified in rodent *TFR1*, mapped to a linear schematic of the TfR1 ectodomain. The amino acid encoded by human *TFR1* at each of these positions is indicated. Residue 109 was also identified as being under positive selection (Table S1). Although potentially of functional relevance, this residue lies outside of the structure of the TfR1 ectodomain and therefore was not analyzed further in the current study. (C) Residue positions under positive selection are indicated in red on the structure of human TfR1 (PDB 1CX8) [65]. TfR1 is a homodimer, and the six sites of positive selection are indicated on the outer edge of each monomer. Known binding regions on TfR1 for Machupo virus GP [5] and MMTV Env [13] are indicated in gray and blue, respectively, and the small region where they overlap is indicated with crosshatching. To the right is shown a rotated view of one edge of the TfR1 dimer.

The entry of viruses into cells is often mediated by specific physical interactions between virus surface proteins and host-encoded cell surface receptors. In the case of the New World arenaviruses, the surface glycoprotein, GP, contacts TfR1 to trigger cellular entry [6]. These viruses infect various rodent species found in the Americas, and each virus has evolved compatibility with the particular TfR1 ortholog encoded by its host species (Figure 1A) [7]–[9]. Several of these viruses, including Junin virus, Machupo virus, and Guanarito virus, have acquired the ability to bind human TfR1 and are currently emerging into human populations through zoonotic transmission [10],[11]. These viruses cause hemorrhagic fevers in humans with case fatality rates of 15–30%, but fortunately, they do not yet spread from human to human efficiently enough to cause large epidemics. Another rodent virus that uses TfR1 for cellular entry is the retrovirus mouse mammary tumor virus (MMTV). The MMTV surface glycoprotein, Env, contacts TfR1 to trigger cellular entry [12]. MMTV infects *Muridae* rodents specifically of the genus *Mus*, including *Mus musculus*, the house mouse (Figure 1A). In contrast to the arenaviruses, MMTV is not known to infect other rodent species or humans. Incompatibility with human TfR1 appears to be the major cellular barrier to zoonosis because MMTV replicates robustly in human cells when receptor-mediated entry is bypassed by transfection of the viral genome directly into cells [13]–[15]. Finally, in carnivores, parvoviruses also bind TfR1 for cellular entry [16]. Canine parvovirus serves as one of the most important models for disease emergence in the wild, as this virus first came into existence in the 1970s when a virus was passed to dogs from another carnivore species [17]. This event centered around viral evolution for compatibility with the dog TfR1 ortholog [18],[19]. Thus, in all three of the virus families that use TfR1, existing evidence suggests that the ability to enter cells through the TfR1 ortholog of a particular species is a necessary criterion for infection in the wild, and that viral adaptation is often required to utilize the TfR1 of new species.

While infectious disease research has long focused on host antiviral proteins, host proteins that facilitate viral replication are now an exploding area of inquiry [20]. These proteins represent novel targets for the development of antiviral drugs because interruption of the interactions between virions and host proteins like TfR1 are predicted to block viral replication. In nature, evolution has utilized two paradigms for achieving this same goal. In some cases, host genes encoding pathogen entry receptors have accumulated promoter or other mutations that cause reduced or no expression of the receptor protein [21]–[27]. However, *TFR1*, given the essential nature of its housekeeping functions, would be unlikely to tolerate hypomorphic mutations. For retroviruses, host genomes are known to employ a second mechanism to block virus entry, one that exploits a unique property of the retroviral lifecycle. Unlike other viruses, retroviruses permanently integrate into the host genome during viral replication. If viral genomes become integrated in the host germline, they can be passed to future generations and inherited in a Mendelian fashion [28],[29]. In several instances, retroviral surface proteins (Envs) expressed from these integrated retroviral copies compete with exogenous viruses for receptor use [30]–[35]. Host genomes are presumably selected to keep these retroviral *env* open reading frames intact because they offer protection against infection by exogenous viruses that use the same receptor [28],[29],[36]. Given the critical role of TfR1 in iron homeostasis, there may be a fitness cost to competitive binding by genome-encoded copies of the retroviral Env. Indeed, there is no evidence for either of these models (hypomorphic mutations or competitive inhibition) in the *TFR1* literature. How, then, do critical genes like *TFR1* respond to virus-driven selective pressure?

Most of what is known about the evolutionary dynamics between host and virus genomes comes from studies of antiviral genes, particularly those encoding viral sensors. Viral sensors (also referred to as “pattern recognition receptors” or “restriction factors”) are host proteins like RIG-I and TRIM5α that recognize and destroy viruses that are attempting to replicate inside of host cells [37],[38]. Because these sensors can be so effective, viruses often encode proteins that antagonize them or their downstream executors [39],[40]. Host genomes are continually selected to encode sensors that better recognize viruses, and viruses are continually selected to evade or disrupt these sensors [41]–[50]. This ongoing evolutionary struggle is called a molecular “arms race” (reviewed in [51]–[53]). Arms races play out in the protein–protein interactions that exist between host and virus proteins, and they drive endless rounds of “positive selection” for mutations that alter these interactions. This results in the rapid evolution of both proteins (host and virus) engaged in the conflict. Indeed, host-encoded viral sensors are often exceptionally genetically divergent between species and diverse within species [41]–[50],[54]–[58]. As a result, such genes are appreciated as major genetic barriers to host switching by viruses in nature, because unique virus mutations are required to counteract the divergent viral sensors present in each new host species [43],[59]–[61].

Arms races have not traditionally been documented in important housekeeping genes. Here, we document recurrent positive selection in rodent *TFR1* and demonstrate that both the protein sequence and the interaction specificities of this receptor are far from static. Using a small evolutionary dataset consisting of *TFR1* gene sequences from only seven rodent species, we identify specific codons in *TFR1* that have been repeatedly targeted by positive selection for amino acid replacement. We find that these rapidly evolving positions correlate to the surfaces on TfR1 that mediate interaction with the two rodent viruses that bind this receptor. We demonstrate experimentally that mutations at these specific receptor residues are potent at altering interactions with virions while not altering receptor expression or function. We show that this evolutionary scenario has driven genetic divergence at this receptor locus that now enforces species barriers to viral transmission. We address the implications of these findings for human TfR1 and identify a human SNP that conveys some protection against cellular entry of a zoonotic rodent arenavirus. Our study demonstrates that the influence of viral pathogens on mammalian genomes goes well beyond the shaping of antiviral genes, as we can now appreciate that even the sequence of important housekeeping genes can be shaped by unremitting antagonism by viruses. However, in this case, collateral damage to cellular functions must be carefully controlled as the evolutionary battle with viruses plays out.

## Results

### Rodent *TFR1* Has Been Subject to Multiple Rounds of Positive Selection for Amino Acid Substitution

We investigated the evolution of *TFR1* in rodents, where two different virus families use this receptor for cellular entry. The type of selective pressure that has acted on a gene can be inferred from the pattern of mutations that it has accumulated over time [62],[63]. The rate at which mammalian genes accumulate amino acid–altering DNA mutations (dN; nonsynonymous mutations) is typically far slower than the rate at which they accumulate mutations that leave the amino acid unchanged (dS; synonymous mutations) [51]. This is because most amino acid–altering mutations are deleterious. This signature (dN/dS<<1) stands in contrast to the pattern that is observed when genes have experienced multiple rounds of positive selection for protein-altering mutations (dN/dS>1). However, in host-virus arms race situations, patterns of dN/dS>1 would not be expected throughout the entire length of a gene, but rather specifically in the codons correlating to the interaction interface between host and virus proteins (reviewed in [51],[52]). We used the codeml program in PAML [64] to analyze dN/dS ratios in codons in an alignment of *TFR1* from seven rodent species, five of which are known host species for the New World arenaviruses or MMTV (Figure 1A). We found variable patterns of codon evolution in *TFR1*. For instance, in codon model M2a, maximum likelihood estimation indicates that 78% of codons are extremely conserved with dN/dS = 0.09, 19% evolve neutrally with dN/dS = 1, and 2.4% are under positive selection with dN/dS = 4.2. Codon models that allow a subset of codons to evolve under positive selection (dN/dS>1) fit the data significantly better than models where positive selection is not allowed (*p*<0.001; Table S1). Thus, while much of the protein sequence of TfR1 is extremely conserved, a small percentage of residue positions are rapidly evolving.

### Patterns of Molecular Evolution Are Consistent with Host-Virus Arms Race Dynamics

The crystal structure of the TfR1 ectodomain has been solved [65]. Six codons that correspond to residues in this structure were assigned to the dN/dS>1 site class with a high posterior probability: K205, L209, N215, S296, T569, and E575 (Table S1). While discontinuous on the linear polypeptide (Figure 1B), the residues corresponding to these codons are located on a single ridge trailing down the outer edge of each monomer of the human TfR1 dimer (red residues in Figure 1C). Remarkably, all of these sites map precisely to the two known virus-binding surfaces on TfR1. Three of these rapidly evolving residue positions (K205, L209, and N215) map to the arenavirus binding surface of TfR1 (gray residues in Figure 1C) [5]. The other three rapidly evolving residues (S296, T569, and E575) fall directly in the surface of TfR1 that binds MMTV (blue residues in Figure 1C) [13]. We hypothesized that rodent *TFR1* is subject to not just one but two different host-virus arms races.

**Figure 2:**
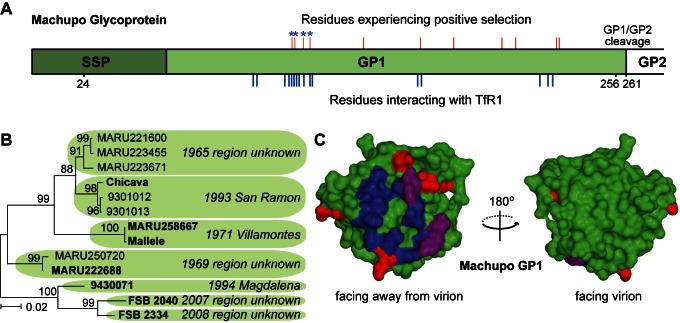
Residues under positive selection on the receptor binding surface of Machupo virus GP1. (A) A diagram of the Machupo virus surface glycoprotein precursor protein, GP. This protein is cleaved into three subunits: the stable signal peptide (SSP), the receptor-binding component GP1, and a transmembrane component GP2. The 16 residue positions that directly contact TfR1, as defined previously [5], are shown with blue lines positioned at the bottom of the diagram. An alignment of codons 24–256, spanning part of the SSP and almost all of GP1, was analyzed for codons with dN/dS>1. Residues corresponding to dN/dS>1 codons are indicated with red lines positioned above the diagram. Asterisks indicate four residues that both directly contact the receptor and are under positive selection. (B) A maximum likelihood tree of the 13 Machupo virus sequences analyzed. All of these viruses were isolated in Bolivia, in the years and regions indicated, from either humans (bold sequences) or *Calomys* mice (other sequences). The tree is unrooted. (C) The crystal structure of Machupo GP1 (PDB 2WFO) [66] showing residues that contact TfR1 [5] (blue), residues under positive selection (red), and the four residues that both contact TfR1 and are under positive selection (purple).

Arms races are predicted to drive positive selection in both the host and virus genes involved, so we next analyzed the gene encoding the arenavirus surface protein, GP, for signatures of positive selection. Because the co-crystal structure has been solved of the Machupo virus surface glycoprotein subunit GP1 in complex with TfR1, the specific residues on GP1 that interact with TfR1 are known (blue lines below protein schematic in Figure 2A). We analyzed an alignment of *gp1* from 13 human and mouse isolates of Machupo virus (Figure 2B). In this alignment, 11 codons bear the signature of dN/dS>1 (red lines above diagram in Figure 2A and Table S2). Ten of these correspond to surface-exposed residues in the GP1 structure [66]. Strikingly, all 10 are located on the surface of GP1 that faces TfR1, and none fall on the opposite side of GP1 that faces the virion (Figure 2C). Four of the residues under positive selection directly contact TfR1, and the rest are located near residues that do (Figure 2C). Using a permutation test, we find that the 16 TfR1-binding residues of GP1 are significantly enriched for sites of positive selection (*p*<0.005). Like all virus surface proteins, GP1 will have also experienced selection for immune escape, a complication that makes signatures of dN/dS>1 more difficult to interpret in viral genes than in host genes. However, GP1 residues in direct contact with TfR1 are unlikely to successfully mutate for the purpose of immune escape during an active infection. An arms race between rodent TfR1 and arenavirus GP1 is thus supported by the rapid evolution of each partner in this interaction, specifically in residues that are known to mediate contact with the other.

**Figure 3:**
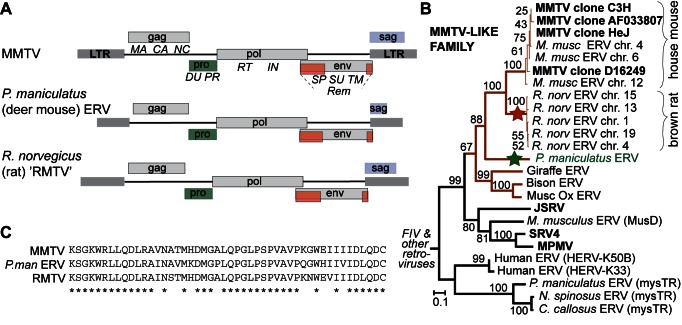
Fossil MMTV-like endogenous retroviruses (ERVs) identified in divergent rodent taxa. (A) A diagram of the MMTV genome is shown, with genes drawn on three levels to indicate different reading frames. Proteins produced from each gene are listed underneath. An MMTV-like ERV was found in the genome of the deer mouse, *Peromyscus maniculatus*. Five MMTV-like ERVs are also evident in the genome of brown rat, *R. norvegicus*, as has previously been noted [67]. Multiple rat ERV copies allowed us to construct the consensus sequence approximating the sequence of the exogenous rat virus (“RMTV”) that gave rise to these ERVs. Genetic distances between each rat ERV and the RMTV consensus (0.008–0.018 substitutions/site), combined with the neutral substitution rate observed in the rat genome (0.00506 substitutions/site/MY) [91], support an RMTV infection of rats that lasted from 3.6 to 1.6 million years ago. A diagram of the RMTV genome is also shown. LTR, long terminal repeat. (B) A beta-retrovirus phylogeny constructed from an alignment of approximately 900 nucleotides in the region of *pro-pol*. In bold are exogenous viral sequences. All others are endogenous viral sequences found integrated in the genomes of the indicated host species. The tree shows that the brown rat and deer mouse (green star) ERVs discussed in the text are more closely related to MMTV than any other virus reported in GenBank. The predicted position of the ancestral RMTV virus is shown (red star). The red branches indicate a family of viruses that we refer to as MMTV-like viruses. A maximum likelihood tree is shown. On each node are bootstrap values, given as percentage of 1,000 replicates. The tree was rooted with feline immunodeficiency virus (FIV), a lentivirus that is not in the beta-retrovirus family. JSRV, Jaagsiekte sheep retrovirus; SRV4, Simian retrovirus 4; MPMV, Mason-Pfizer monkey virus. See also Figure S1. (C) A small portion of the aligned Pol protein translation is shown to demonstrate the degree of sequence similarity between the three MMTV-like viruses discussed.

In the *TFR1* dataset analyzed, only one of the rodent species included is known to harbor MMTV in the wild (house mouse; Figure 1A). It was thus unclear why we detected positive selection in the MMTV binding surface of TfR1 with the rodent dataset that was used. We hypothesized that either the evolutionary signature in the MMTV binding surface of TfR1 was driven by something else, or that MMTV-like viruses once circulated more widely through rodent genera. We reasoned that if the latter hypothesis is true, “fossils” of these extinct viruses might be found in the form of endogenous retroviruses (ERVs) integrated into the genomes of their former host species. Indeed, we identified MMTV-like ERVs in the genomes of the brown rat (*Rattus norvegicus*) and the North American deer mouse (*Peromyscus maniculatus*) (Figure 3 and Figure S1). The full-length ERV identified in the deer mouse genome is particularly interesting because this rodent is in the same family as the arenavirus host species (*Cricetidae*; Figure 1A). These ERVs reveal that MMTV-like viruses once circulated more widely amongst rodents, supporting the model that rodent *TFR1* may have experienced selection imposed by these viruses. Interestingly, MMTV appears to be a virus in retreat, with a shrinking host range. We cannot exclude the possibility that MMTV-like viruses still infect other rodent species and have simply not been identified, but such viruses have not been reported in the literature or in GenBank [67], and are absent from large metagenomic surveys of rodent feces [68]. These MMTV ERVs are thus reminiscent of the many ERV families found in the human genome, none of which currently circulate in infectious form [28].

**Figure 4:**
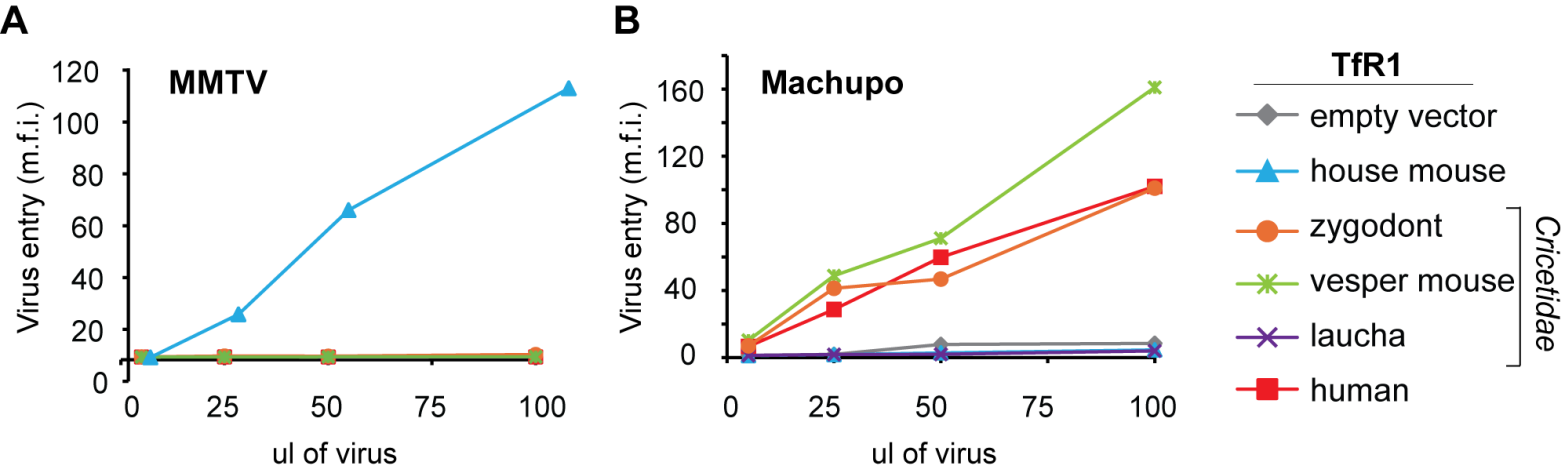
Cellular entry of MMTV and Machupo virus is permitted by some but not all rodent TfR1 orthologs. MDCK cells were transduced to stably express the TfR1 of various *Cricetidae* and *Muridae* species used in the evolutionary analysis, or human TfR1. An extracellular FLAG tag was added to each receptor and cell surface expression was monitored on live cells by flow cytometry. These cells were infected with GFP-encoding retroviral vectors pseudotyped with the surface glycoproteins of (A) MMTV or (B) Machupo virus. Relative entry is scored by the mean fluorescent intensity (m.f.i.) of GFP. As previously reported, the surface protein of Machupo virus mediates cellular entry through the TfR1 of the Machupo virus host species, the vesper mouse (green line), and to a lesser extent through TfR1 of zygodont (orange line) and human TfR1 (red line) [7]. In contrast, cellular entry of MMTV was supported strictly by the TfR1 of house mouse.

Based on these findings, TfR1 may have experienced high levels of sequence divergence on the MMTV-binding surface due to selection for mutations that blocked entry by these MMTV-like viruses. Consistent with this, we find that TfR1 orthologs from three different *Cricetidae* species are highly recalcitrant to entry by MMTV (Figure 4), even though this rodent family appears to once have harbored a similar virus. In an arms race between TfR1 and MMTV, the MMTV Env should also be evolving in response to the evolution of TfR1. Compared to Machupo virus GP1, far less is known about the amino acids in MMTV Env that bind to TfR1, as there is no co-crystal structure of Env in complex with TfR1. However, a five amino acid receptor binding motif in MMTV Env has been identified [69]. We find that this motif has a distinct protein sequence depending on the particular rodent host species from which each virus was isolated (Figure S2), consistent with viruses having uniquely evolved compatibility with each host TfR1 (before they potentially went extinct). An incomplete understanding of receptor binding determinants in MMTV Env, and the fact that most of these viruses now exist as endogenous copies, make it difficult to draw specific conclusions about the evolution of MMTV Env. Nonetheless, an arms race between TfR1 and MMTV is supported by the rapid evolution of residues on the MMTV-interaction surface of TfR1, the discovery that MMTV-like viruses once infected rodents more broadly providing a model for what drove this selection, and the observation that several *Cricetidae* TfR1 in their current form do not support MMTV entry, suggesting that they could have been selected for this property.

### Mutations at Sites of Positive Selection Modulate Virus Entry Without Affecting TfR1 Function

**Figure 5:**
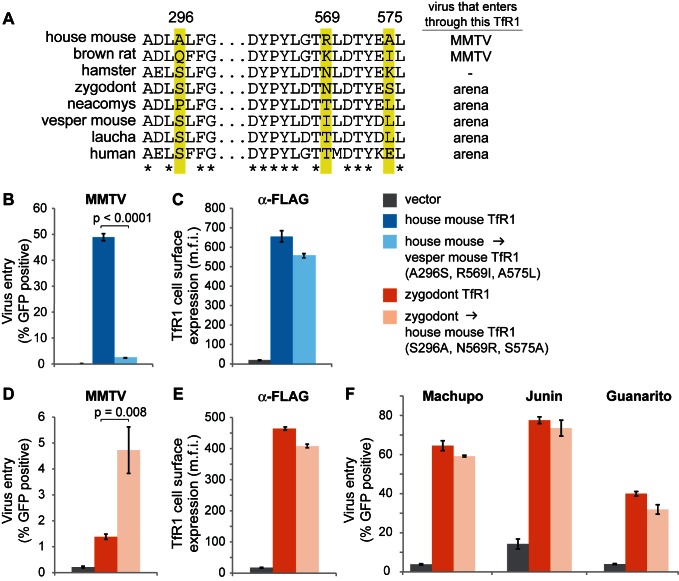
Mutations at sites of positive selection alter MMTV entry through TfR1. (A) A partial TfR1 sequence alignment shows the three residue positions under positive selection (highlighted in yellow) located in the MMTV binding region. Asterisks indicate completely conserved residue positions, while positions under positive selection are highly variable. The viruses that have been previously shown to enter cells via each of these receptors are also summarized (although in the case of brown rat, cellular entry of MMTV via the rat TfR1 does not lead to productive infection [13], consistent with TfR1 usage being a necessary but not sufficient determinant of host range in the wild). In the remaining panels, amino acids are swapped between the species indicated at these three positions under positive selection. In one case (blue graphs), these three positions in the house mouse TfR1 were altered to encode the amino acids found in the vesper mouse TfR1. In the second case (orange graphs), these three positions in the zygodont TfR1 were altered to encode the amino acids found in the house mouse TfR1. (B and D) MDCK cells stably expressing the indicated TfR1-FLAG were infected with GFP-encoding retroviral vectors pseudotyped with the surface glycoprotein of MMTV. Virus entry was scored by measuring the percentage of GFP positive cells using flow cytometry. (C and E) Cell surface expression of TfR1 (mean fluorescent intensity) measured on live cells with a fluorescently labeled α-FLAG antibody. (F) Cellular entry of retroviral vectors pseudotyped with the surface glycoproteins of three different arenaviruses (Machupo, Junin, and Guanarito). In all experiments, three replicates were performed and error bars indicate one standard deviation.

**Figure 6:**
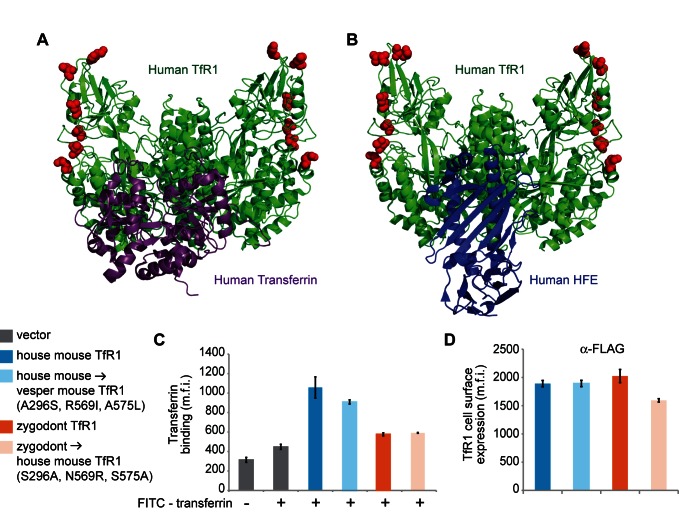
Mutations at sites of positive selection do not alter TfR1 association with host proteins. Co-crystal structures of human TfR1 in complex with (A) human transferrin (1SUV) [4] and (B) human HFE (1DE4) [2] illustrate that sites of positive selection (red) fall at a distance from these protein–protein interaction surfaces. Thus, mutations at these sites are not predicted to affect important host-beneficial functions of TfR1. MDCK cells stably expressing wild-type and mutant TfR1 were incubated with media containing FITC-labeled iron-loaded mouse transferrin. The cells were then washed and analyzed by flow cytometry for the mean fluorescent intensities (m.f.i.) of (C) FITC-transferrin and (D) a fluorescently labeled α-FLAG antibody measuring TfR1 surface expression. In all experiments, three replicates were performed and error bars indicate one standard deviation.

To test this MMTV resistance hypothesis further, we simulated the evolution of an MMTV-resistant receptor by mutating only the residue positions under positive selection in the MMTV binding surface (Figure 5A). We mutated the TfR1 of house mouse, the MMTV host, so that these three positions now encode the amino acids found in the TfR1 of the vesper mouse, which is not susceptible to MMTV. MDCK (dog) cells were transduced to stably express the mutant or wild-type TfR1 protein. These cells were chosen because dog TfR1 does not support entry by arenaviruses [9] or MMTV [13]. An extracellular FLAG tag was added to each receptor so that cell surface expression could be monitored on live cells by flow cytometry. We then measured the cellular entry of GFP-encoding retroviral vectors expressing the MMTV Env on their surface (MMTV pseudoviruses). Indeed, the three mutations in house mouse TfR1 almost completely abolished the entry of MMTV into cells (Welch t-test, *p*<0.0001, one-tailed; Figure 5B) without significantly altering receptor cell surface expression (Figure 5C). None of the sites of positive selection that we identified are found near the dimerization domain of TfR1, the region known to be most important for interaction with iron-transport binding partners (Figure 6A,B) [2]–[4],[70]. We confirmed that these mutations indeed do not alter transferrin binding (Figure 6C,D). Thus, amino acid substitutions at these sites in TfR1 can block virus entry without deleterious consequences to surface expression or receptor function, providing a clear hypothesis for why they might have a strong selectable advantage in MMTV-infected rodent populations.

If positively selected residues are key modulators of virus compatibility, we reasoned that mutations at these sites should also render MMTV-resistant TfR1s susceptible to MMTV entry. Because species divergence can lead to subtle structural differences in receptors, creating a gain-of-function phenotype with just three amino acid changes should be substantially more difficult than creating a loss of function phenotype in a receptor where virus-binding is currently intact. Nonetheless, mutating the three positively selected residues in the MMTV binding surface of zygodont TfR1 to match the corresponding residues found in TfR1 of house mouse (the MMTV host) led to a significant increase in MMTV entry (Welch t-test, *p* = 0.008, one-tailed; Figure 5D) without enhancing cell-surface expression (Figure 5E), transferrin binding (Figure 6C,D), or entry of three arenaviruses (Figure 5F). Thus, we have shown that swapping amino acids encoded at positively selected sites can swap virus-susceptibility phenotypes of TfR1 in both a gain-of-function and loss-of-function manner. Mutations at just three residue positions acutely regulate virus entry while preserving receptor expression and transferrin binding for the host.

### Is Human *TFR1* Poised for an Arms Race with Zoonotic Arenaviruses?

Every round of positive selection of the rodent *TFR1* gene began with a random mutation that arose in a single rodent individual. If this mutation offered protection against virus entry while not otherwise causing major fitness defects related to iron homeostasis, it would have been favored by natural selection and would have become more common or even fixed in the population where it arose. Because the New World arenaviruses are currently emerging into human populations, they are now beginning to exert selective pressure on the human population as well. For instance, there have been approximately 30,000 cases of Argentine hemorrhagic fever caused by the Junin virus since the 1950s, with a case fatality rate of 20% [11]. The geographic region at risk for this disease is expanding into north-central Argentina, and currently includes an area populated by around 5 million people [11]. Individuals with genotypes that make them less susceptible to infection or severe illness are expected to survive with bias over other individuals. This selection would intensify as the frequency or severity of the disease increases. In such cases, natural selection would be expected to act at any genetic locus where functionally distinct alleles exist within the human population. We wished to investigate whether *TFR1* may be one such locus.

**Figure 7:**
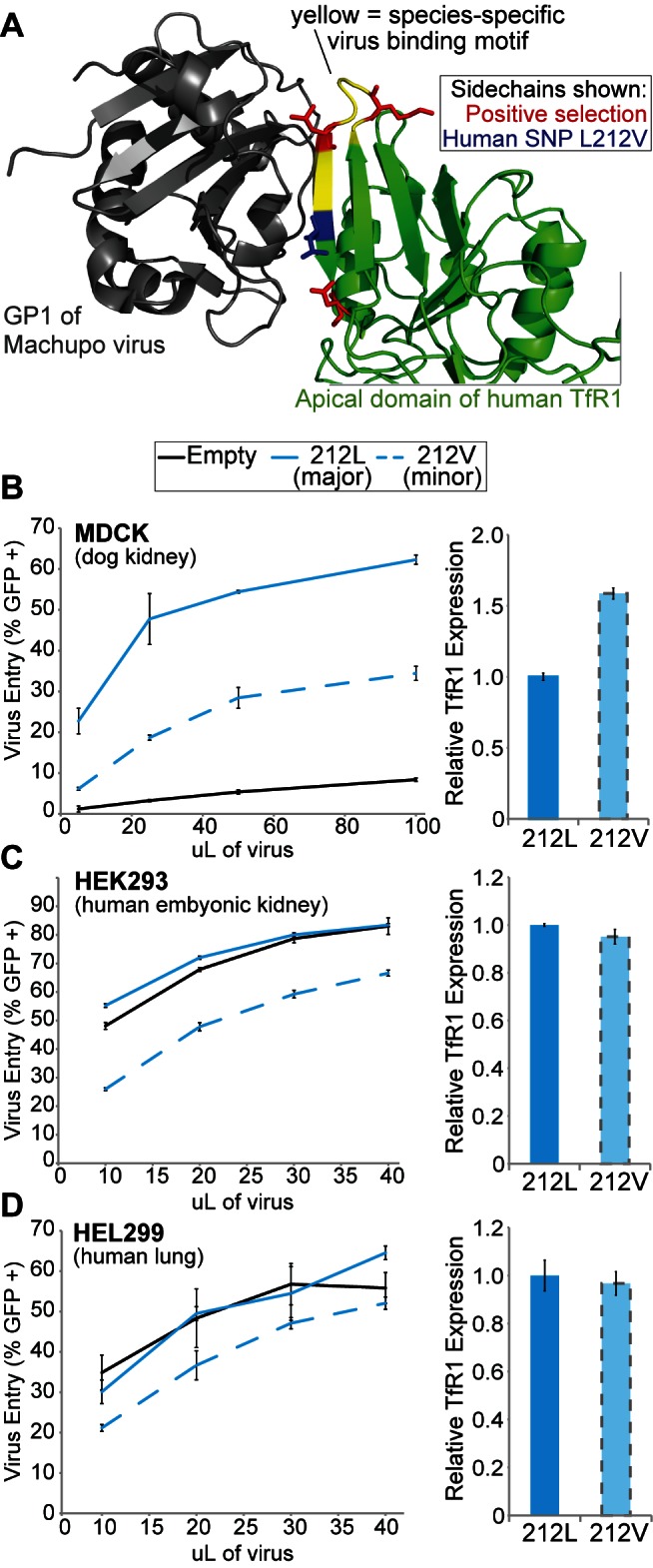
A human SNP in TfR1 is protective against Machupo virus entry. (A) The apical domain of human TfR1 is shown in green, in a co-crystal structure with the Machupo GP1 shown in grey (PDB 3KAS) [5]. The βII-1–βII-2 species-specific virus binding motif (residues 204–212) is highlighted in yellow. The side chains of residue positions identified as evolving under positive selection are shown in red. A human SNP (L212V; rs41301381) has been reported at position 212, shown in blue. (B) MDCK, (C) HEK293, or (D) HEL299 cells were stably transduced to express either 212L TfR1 or 212V TfR1 (or an empty vector) and then infected with various amounts of Machupo pseudovirus. Virus entry is scored by percentage of cells that become GFP positive (+). The error bars in the HEL299 experiment are large due to difficulty in sorting these cells. Nonetheless, this pattern of relative entry between the different *TFR1* alleles expressed in HEL299 cells was observed in four independent experiments (not shown). (B, C, D, right-hand panels) Relative cell-surface expression of human TfR1 variants in each cell line was measured on live cells with a fluorescently labeled α-FLAG antibody. In all experiments, three replicates were performed and error bars indicate one standard deviation.

TfR1 interacts with arenaviruses and MMTV through distinct interaction surfaces (Figure 1C). TfR1 is 760 amino acids long, but a small stretch of nine residues from 204 to 212 is the major determinant of species-specificity for arenavirus entry (colored yellow in Figure 7A). These residues span two beta strands and the intervening loop (βII-1–βII-2). Two of the sites of positive selection (residues 205 and 209) fall in this stretch of nine residues, and the third (residue 215) falls three amino acids away (colored red in Figure 7A). As we demonstrated for the sites under positive selection in the MMTV binding surface, the introduction of amino acids from different rodent species at positions in this stretch has been previously shown to alter patterns of virus compatibility [7],[8]. Additionally, substitution of rodent-encoded amino acids at these residues can convert human TfR1 into an entry receptor for currently non-zoonotic rodent arenaviruses [5],[8]. By querying SNP databases, we identified a human SNP located in this structural feature, L212V (colored blue in Figure 7A). Because of the localization of this SNP near the residues under positive selection, we hypothesized that the L212V human polymorphism might affect arenavirus entry.

To test this, we again focused on Machupo virus. We constructed stable cell lines that express either human 212L or 212V TfR1. In the context of MDCK cells, dog TfR1 does not allow entry by Machupo virus, so the expression of either human allele allows more virus entry than is observed in MDCK cells alone (Figure 7B). However, the minor TfR1 212V variant supports about half the level of entry as seen with TfR1 212L (Figure 7B). Valine at position 212 may lead to a modest decrease in binding affinity with GP1 due to loss of a hydrophobic contact, based on the observation that two residues of Machupo GP1 (Phe226 and Pro223) are in van der Waals contact with TfR1 Leu212 [5]. We next stably expressed the human 212V and 212L *TFR1* alleles in human cell lines that are themselves homozygous for 212L: HEK293 (kidney) and HEL299 (lung). Lung cells are especially relevant since arenaviruses are transmitted to humans through respiratory inhalation. In both cases, expression of the minor 212V allele was again protective against virus entry compared to the wild-type allele (Figure 7C,D). Thus, we have identified a SNP (L212V) that conveys some protection against arenavirus entry, at least in vitro.

The L212V SNP has only been reported in Asian populations (Chinese and Japanese), while TfR1-utilizing arenaviruses have only been found in the Americas. We sequenced *TFR1* from 18 indigenous Central and South American individuals, but identified no instances of this polymorphism. Like all SNPs, this SNP arose randomly and may have no fitness advantage or disadvantage in the Asian populations where it is found, since TfR1-utilizing arenaviruses are not found in that part of the world. Nonetheless, this SNP could begin to experience selection if the rodent populations that carry these viruses were introduced into Asia, if these arenaviruses ever evolved to spread efficiently from human to human, or in the event of an intentional release of these viruses [71]. The data shown in Figure 7C,D indicate that protective *TFR1* alleles can act in a semidominant fashion with regards to virus entry, because the human cells used in these experiments also express wild-type TfR1. We speculate that this occurs either because mutant and wild-type TfR1 proteins are forming heterodimers with one another, or because expression of a second allele that is functional for iron-uptake results in lower levels of wild-type TfR1 (*TFR1* expression levels are tightly regulated for the purpose of maintaining iron homeostasis [10]). Either model would also be relevant in heterozygous individuals, suggesting that selection could act on SNPs conveying protection against viral entry even when they are rare and found predominantly in heterozygotes.

## Discussion

In this study we show that the protein sequence and interaction specificities of rodent TfR1 have been dynamic over time, shaped by selective pressures imposed by viruses. These dynamics have played out through mutations accumulated at just a small number of residue sites, where mutations decrease virus entry without measurably affecting receptor expression or iron-transport functions. *TFR1* represents the first case, to our knowledge, where the evolution of a single host gene is driven by two host-virus arms races at once. In the case of the MMTV binding surface, this has played out through three residue positions coordinated in three-dimensional space. In the arenavirus binding surface, the target of selection has been a small surface-exposed structural feature, in which we were able to detect positive selection of three of the residues. Outside of rodents, TfR1 is used by a third family of viruses, the parvoviruses, and carnivore *TFR1* is also under positive selection [72]. *TFR1* evolution has thus been shaped by viruses in two separate species groups (rodents and carnivores) and by every viral pathogen known to use this receptor. These findings now explain how *TFR1* became divergent enough to create species-specific interactions with all three of these virus families. If even a few residue positions can evolve to block virus entry without collateral damage to cellular function, host-virus arms race dynamics can unfold even in genes encoding highly conserved and essential housekeeping proteins.

This evolution of *TFR1* can be put into contrast with other types of pathogen-driven positive selection of host genes. The human *CCR5* gene encodes a co-receptor for HIV cellular entry. Some humans encode a variant allele of *CCR5*, *CCR5Δ32*, where a 32 base pair deletion gives rise to a defective receptor that is not expressed on the cell surface [73]. Individuals homozygous for this allele are almost completely resistant to HIV infection, and even heterozygous genotypes afford some protection due to reduced expression of wild-type CCR5. Like the model proposed herein for *TFR1* L212V, *CCR5Δ32* pre-dates HIV and may or may not have had any functional significance before the HIV pandemic. Nonetheless, it has become highly relevant in a world with HIV/AIDS. Like HIV, most simian immunodeficiency virus (SIV) strains also use CCR5 as a co-receptor. In a fascinating case of convergent evolution, some sooty mangabeys and red-capped mangabeys also encode null or defective alleles of *CCR5* [21],[23]. Similarly, the *DARC* gene encodes a chemokine receptor that is used as an entry receptor by some malaria-causing *Plasmodium* species. A cis-regulatory polymorphism that silences *DARC* expression in erythrocytes has arisen independently in human populations from different parts of the world and is highly protective against *Plasmodium vivax* and *Plasmodium knowlesi* infection [24],[25]. Similar mutations have arisen in the cis-regulatory region of *DARC* in African baboons, and these are associated with resistance to a malaria-like parasite common in baboon populations [26]. In all of these cases, it has been speculated that selective pressure exerted by pathogens has driven these hypomorphic receptor alleles to high frequency in the affected human and nonhuman primate populations.

These *CCR5* and *DARC* examples represent a more common mode of pathogen-driven positive selection (not recurrent) than the one demonstrated for *TFR1*, and there are several important differences. When receptor genes experience hypomorphic mutations, the predominant evolutionary strategy available to viruses will be to use a new receptor altogether. Indeed, the SIV strains that infect sooty and red-capped mangabeys (SIVsmm and SIVrcm) have both evolved to use alternate co-receptors [21],[23]. A few *CCR5Δ32* homozygous humans have also been reported to be infected with HIV, again through mutations that allow the virus to use an alternate co-receptor (CXCR4 in this case). Hypomorphic mutations in receptors are not expected to be “serially replaced” due to arms race dynamics. Rather, viral evolution to use a new receptor ends the arms race with the original receptor gene and starts a new one with the new receptor gene. The *CCR5* and *DARC* examples also involve evolutionary time scales millions of years shorter than what has been demonstrated in the current study; because these hypomorphic alleles are circulating in populations of individuals and are not shared between species, they have arisen relatively recently. Also, because these mutations simply reduce cellular expression of the encoded receptors, they presumably have some negative fitness effect on the host. The TfR1 example that we provide here is unique because solutions to viral entry have been found that appear to lack collateral damage to transferrin binding, and presumably to other host functions as well. Because of this, these mutations become common or fixed in populations where they occur, and are serially replaced as viruses continue to evolve and as rodents continue to speciate.

There is reason to believe that host-virus arms races are also shaping the protein sequence of other virus entry receptors in the manner described here. There are several other examples where significant sequence and functional divergence exist both on the side of a virus and its host entry receptor. For instance, certain strains of murine leukemia virus (MLV) use the rodent XPR1 receptor for cellular entry [74]. There are several functionally distinct variants of the *XPR1* gene encoded by rodents of the genus *Mus*, each with its own pattern of virus susceptibilities. The viruses that use this receptor are also highly variable in the receptor-binding portion of their surface protein, Env. High levels of sequence divergence and disparate interaction specificities have also been observed between the entry receptor TVB encoded by birds and the avian leukosis virus (ALV) strains that use this receptor [75]. In neither of these cases is the housekeeping function or structure of the receptor known, so the pleiotropic consequences of pathogen-driven selection remain to be explored. However, both of these viruses can evolve to use new allelic forms of their receptor encoded by new hosts, suggesting that the receptors are important determinants of host range. High levels of sequence divergence, along with polymorphic and species-specific interactions between receptors and viruses, should be the hallmark for this type of evolution. These patterns have also been observed in other pairs of receptors and viruses [72],[76]–[80], suggesting that arms races might shape many receptors and potentially other types of housekeeping proteins exploited by viruses as well [81],[82].

Traditionally, TfR1 has been viewed as a housekeeping protein with an immensely important and conserved role in the cell. This study provides a much richer understanding of the multiple dynamic roles that this receptor is balancing in nature.

## Materials and Methods

### Codon-Based Analysis of Molecular Evolution

Rodent *TFR1* and Machupo *gp1* sequences were analyzed for positive selection. Database accession numbers for sequences used are listed in Tables S1 and S2. Sequences were aligned in Clustal [83], with minor adjustments made by hand (these two alignments contain few or no indels, respectively). jModeltest v2.1.1 [84] was used to select the best-fit model of nucleotide substitution, which was HKY+G in both cases. Phylogenetic trees for each sequence set were built by the maximum likelihood method implemented in MEGA5 [85]. The *TFR1* gene tree matches the species tree of these rodents [86]. Because the Machupo *gp1* sequences represent viral isolates from the same population, GARD [87] was run on the *gp1* alignment to confirm the lack of phylogenetic breakpoints indicative of recombination. For both datasets, maximum likelihood analysis of dN/dS was then performed with codeml in the PAML 4.1 [64] software package. To detect selection, multiple alignments were fit to the NSsites models M1a (neutral model, codon values of dN/dS are fit into two site classes, one with value between 0 and 1, and one fixed at dN/dS = 1), M2a (positive selection model, similar to M1a but with an extra codon class of dN/dS>1 allowed), M7 (neutral model, codon values of dN/dS fit to a beta distribution, dN/dS>1 disallowed), M8a (neutral model, similar to M7 except with a fixed codon class at dN/dS = 1), and M8 (positive selection model, similar to M7 but with an extra class of dN/dS>1 allowed). Model fitting was performed with multiple seed values for dN/dS (ω) and assuming either the f61 or f3x4 model of codon frequencies [88]. Likelihood ratio tests were performed to assess whether permitting some codons to evolve under positive selection gives a significantly better fit to the data than models where positive selection is not allowed. The results obtained were shown to be robust to changes in the codon frequency model used, and the seed value for dN/dS (Tables S1 and S2). Posterior probabilities of codons under positive selection in M8 were then inferred using the Naive Empirical Bayes (NEB) algorithm. Coordinates for molecular structures were obtained from the RSCB protein database (http://www.pdb.org/) and rendered using PyMOL (http://www.pymol.org).

### Identification and Phylogenetic Analyses of Fossil Viruses

Full-length MMTV sequences were obtained on GenBank (AF228552, D16249, AF033807, AF228551). These sequences were used to BLAT [89] the current assemblies of the *M. musculus* (mm9) [90] and *R. norvegicus* (rn4) [91] genomes on the UCSC genome browser [92], recovering the indicated ERVs in these genomes. The nr/nt database for rodents (taxid:9989) at NCBI was searched for similar sequences in other species using the discontiguous megablast search algorithm with full-length MMTV as a query, and using the tBLASTx algorithm with MMTV *pol* as a query. Both of these approaches identified the *Peromyscus maniculatus* ERV buried in the sequence of GenBank record EU204642 (a BAC clone containing the deer mouse beta-globin gene cluster). A relatively young age of this ERV can be inferred from the fact that one open reading frame (*pol*) is still uninterrupted, and from the observation that the 5′ and 3′ LTRs differ at only 1 out of 917 positions. The giraffe, bison, and musk ox sequences are from [93]. Exogenous and endogenous beta-retrovirus genome sequences were aligned with MUSCLE [94] as implemented in MEGA5 [85]. jModeltest v2.1.1 [84] was used to select GTR+I+G as the best-fit model of nucleotide substitution. Phylogenetic trees were built by the maximum likelihood method implemented in MEGA5. Positions in which one or more sequences contained a gap were excluded during tree building. One thousand bootstrap replicates were performed and results are presented as percentage of replicates that supported each node.

### Human Variation

The L212V SNP in human *TFR1* (rs41301381) was identified in data deposited by the 1000 Genomes Project (http://browser.1000genomes.org). As of Release 12, L212V had been found as a heterozygous SNP in 11 individuals, with no homozygous carriers identified. Three of these individuals were Han Chinese from the South (CHS population), six were Han Chinese from Beijing (CHB population), and two were Japanese individuals (JPT population). In total, 11 out of 286 Asian individuals surveyed were heterozygous at this position, yielding a genotypic frequency of 0.038 in Asia. This SNP has not been included in the HapMap Genotyping Project (as of Release 28).

### Cells and Plasmids

Human embryonic kidney 293T cells (ATCC CRL-11268), HEK293 cells (ATCC CRL-1573), human embryonic lung HEL299 cells (ATCC CCL-137), and canine kidney MDCK.2 cells (ATCC CRL-2936) were all maintained in Dulbecco modified Eagle's medium (Cellgro) supplemented with 10% fetal bovine serum (Gibco), 100 units ml^−1^ penicillin, 100 µg ml^−1^ streptomycin, and 2 mM L-glutamine (Cellgro). Human, *Mus musculus*, *Calomys musculinus*, *Calomys callosus*, and *Zygodontomys brevicauda TFR1* with an encoded C-terminal FLAG tag were moved from pcDNA3.1 (+) vectors (described previously [7]) into the Gateway entry vector pCR8 using the pCR8/GW/TOPO TA Cloning Kit (Invitrogen). The following primers were used to amplify *TfR1* for TA cloning: 5′-TTAATACGACTCACTATAGGG-3′ and 5′-TAGAAGGCACAGTCGAGGC-3′. Gateway LR recombination (Invitrogen) was performed to transfer *TFR1* genes from pCR8 into the entry site in a Gateway-converted LPCX retroviral vector. Site-directed mutagenesis of the human, *M. musculus*, and *Z. brevicauda TFR1* orthologs was performed using QuikChange Site-Directed Mutagenesis kit (Stratagene). Plasmids encoding Machupo, Junin, and Guanarito GP have been described previously [6]. An MMTV Env-encoding plasmid (pQ61) was kindly provided by Dr. Susan Ross (via Dr. Jackie Dudley).

### Generation of Stable Cell Lines

The above described LPCX:*TFR1* retroviral vectors were packaged in 293T cells by co-transfecting them along with the NB-MLV packaging plasmid pCS2-mGP [95] and pC-VSV-G using Fugene (Roche). Supernatants were collected and used to infect MDCK.2 (dog) cells. After 24 h, media containing 3.5 µg ml^−1^ puromycin was added to select for transduced cells (1.0 µg ml^−1^ puromycin was added when creating the HEK293 and HEL299 stable cell lines). These receptors have a C-terminal FLAG tag that is extracellular when the receptor is at the cell surface [8]. Expression of TfR1 proteins was detected in live cells by flow cytometry using an anti-FLAG antibody conjugated with Allophycocyanin (Abcam, catalog ab72569). Stable cell lines expressing human 212L and 212V *TFR1* alleles were made in MDCK, HEK293, and HEL299 cells as described above.

### Entry Assays

Arenavirus GP or MMTV Env pseudotyped MLV recombinant retroviruses were packaged in 293T cells. Fugene (Roche) was used to co-transfect the GFP-encoding transfer vector pQCXIX (BD Biosciences) along with plasmids encoding MLV Gag-Pol and one of the viral surface glycoproteins Machupo GP, Junin GP, Guanarito GP, or MMTV Env. After 48 h, supernatants containing viruses were harvested, filtered, and frozen at −80°C. For entry assays, cell lines stably expressing various TfR1 orthologs or human alleles were plated at a concentration of 1.0×10^5^ cells per well in a 24-well plate and, after 24 h, infected with pseudotyped virus along with 5 µg ml^−1^ polybrene. The plates were spinoculated with centrifugation at 350*g* for 1.25 h at 30°C. After 2 h of incubation at 37°C, cells were washed once with PBS and the media was replaced. Two days postinfection, cells were analyzed by flow cytometry. Cells were first gated for live cells and then, using an anti-FLAG antibody conjugated with Allophycocyanin (APC; Abcam, catalog ab72569), further gated such that all samples were narrowed to the same log decade of receptor expression (capturing the majority of cells but excluding outliers). Where TfR1 expression levels are reported, this is the mean fluorescent intensity within this gated population (10,000 cells). These same 10,000 cells were scored for expression of GFP (viral entry). Analysis of flow cytometry data was performed using FlowJo 8.8.6 (TreeStar Inc, Ashland, OR).

### Transferrin Binding Assays

MDCK.2 stable cell lines expressing FLAG-tagged TfR1 orthologs were trypsinized and aliquoted in triplicate at a concentration of 2.5×10^5^ cells/tube. The cells were washed with DPBS with 1% ovalbumin (Sigma). The cells were then resuspended in 200 µL of DPBS with 1% ovalbumin containing 1∶500 dilution of FITC-conjugated Mouse transferrin (2.0 mg/mL stock concentration; Jackson ImmunoResearch, 015-090-050) and incubated at 37°C for 60 min. Anti-DDDDK (FLAG) tag antibody conjugated with Allophycocyanin (0.1 mg/mL stock concentration; Abcam, catalog ab72569) was added to the cells at a 1∶100 dilution and incubated on ice for 20 min. The cells were then washed twice, resuspended in DPBS with 1% ovalbumin, and then analyzed by flow cytometry. Cells were first gated for live cells and then further gated such that all samples were narrowed to the same log decade of receptor expression (capturing the majority of cells but excluding outliers). Where TfR1 expression levels are reported, this is the mean fluorescent intensity within this gated population (10,000 cells). These same 10,000 cells were simultaneously analyzed for transferrin binding with FITC. Analysis of flow cytometry data was performed using FlowJo 8.8.6 (TreeStar Inc., Ashland, OR).

## Supporting Information

Figure S1 Phylogenetic analysis of MMTV-like beta-retroviruses. Beta-retrovirus phylogeny constructed from (A) approximately 900 bases in the region of *pro-pol* or (B) for select viruses where full-length sequence was available, approximately 5,000 aligned bases spanning from the middle of *gag* to the end of *pol*. In bold are exogenous viral sequences. All others are endogenous viral sequences found integrated in the genomes of the indicated host species. In both panels, maximum likelihood trees are shown. On each node are bootstrap values, given as percentage of 1,000 replicates. Trees were rooted with FIV (feline immunodeficiency virus), a lentivirus that is not in the beta-retrovirus family. The two human ERVs included here (HERV-K50B and HERV-K33) are the highest scoring HERV matches to MMTV, based on BLAST search scores.
(PDF)

Figure S2 Evolution of the putative receptor binding motif of MMTV Env. A partial alignment of the viral protein Env is shown. The alignment includes all available rodent MMTV and MMTV-like virus sequences, as described in more detail in the manuscript, including the endogenous retrovirus found in the *Peromyscus maniculatus* genome. The TfR1 binding determinants of MMTV Env have not fully been mapped, but a TfR1-binding motif has been described [69] and is shown here in yellow. Changes from the MMTV sequence in this region are shown in bold. Viruses and endogenous retroviruses (ERVs) isolated from each of the three species encode different residues in this motif, but the functional significance of this is unknown.
(PDF)

Table S1 PAML analysis of rodent *TFR1* sequences. This table summarizes the codon-based analysis of dN/dS performed on rodent *TFR1* sequences.
(PDF)

Table S2 PAML analysis of Machupo virus *gp1* sequences. This table summarizes the codon-based analysis of dN/dS performed on Machupo virus *gp1* sequences.
(PDF)

## Abbreviations

ALV: avian leukosis virus
ERV: endogenous retrovirus
GP: glycoprotein
HFE: hereditary hemochromatosis protein
LTR: long terminal repeat
MLV: murine leukemia virus
MMTV: mouse mammary tumor virus
SIV: simian immunodeficiency virus
TfR1: Transferrin Receptor 1
