# Supplementary figures and images for: Dual Host-Virus Arms Races Shape an Essential Housekeeping Protein
Source: PLoS Biol. 2013 May 28;11(5):e1001571. doi: 10.1371/journal.pbio.1001571 (PMC3665890; doi:10.1371/journal.pbio.1001571)

Demogines et al  
Figure S1

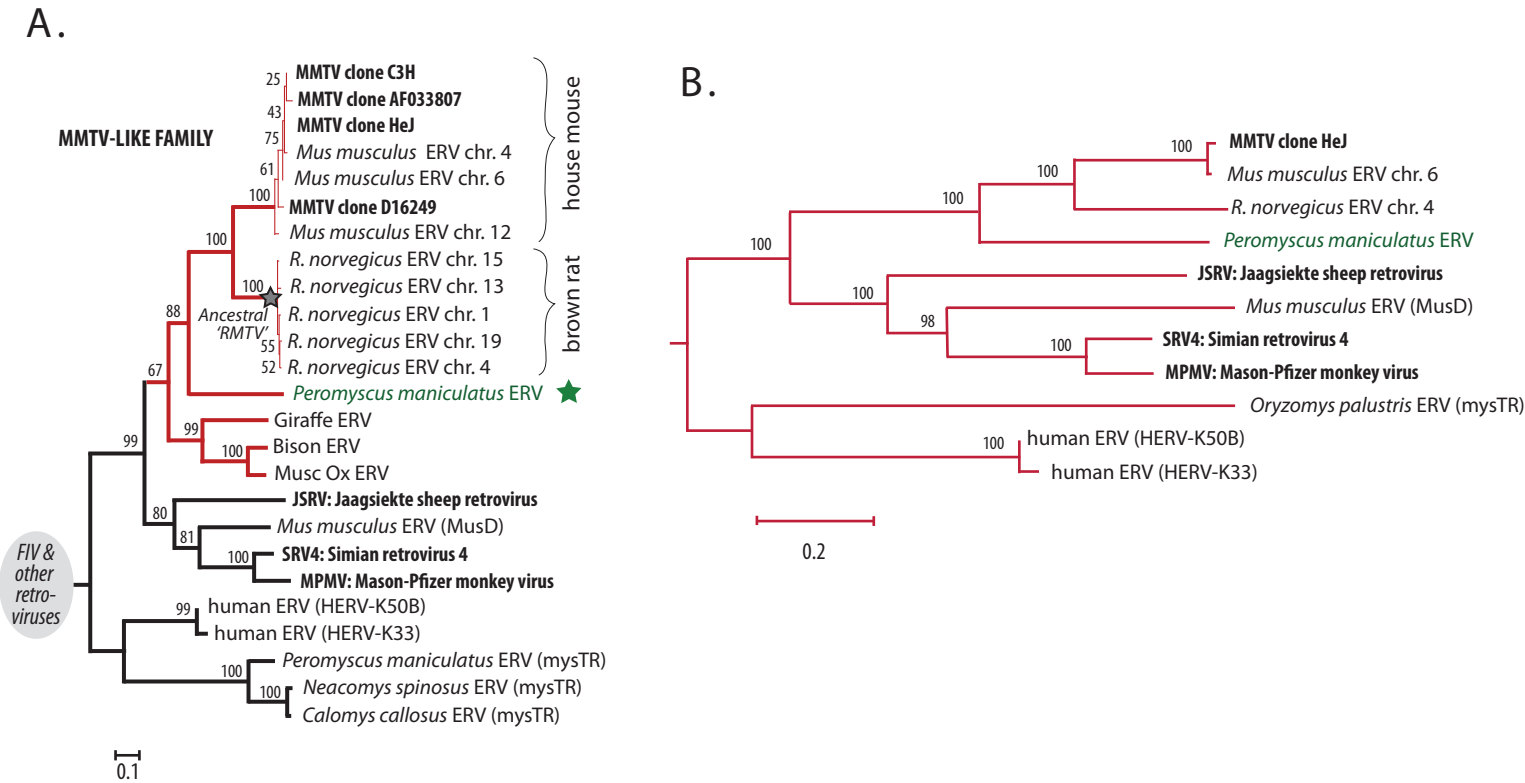

Supplement: Figure S1 — Phylogenetic analysis of MMTV-like beta-retroviruses. Beta-retrovirus phylogeny constructed from (A) approximately 900 bases in the region of pro-pol or (B) for select viruses where full-length sequence was available, approximately 5,000 aligned bases spanning from the middle of gag to the end of pol. In bold are exogenous viral sequences. All others are endogenous viral sequences found integrated in the genomes of the indicated host species. In both panels, maximum likelihood trees are shown. On each node are bootstrap values, given as percentage of 1,000 replicates. Trees were rooted with FIV (feline immunodeficiency virus), a lentivirus that is not in the beta-retrovirus family. The two human ERVs included here (HERV-K50B and HERV-K33) are the highest scoring HERV matches to MMTV, based on BLAST search scores. (PDF) [file pbio.1001571.s001.pdf]
