# Supplementary material for: Dual Host-Virus Arms Races Shape an Essential Housekeeping Protein
Source: PLoS Biol. 2013 May 28;11(5):e1001571. doi: 10.1371/journal.pbio.1001571 (PMC3665890; doi:10.1371/journal.pbio.1001571)
Supplement: Figure S2 — Evolution of the putative receptor binding motif of MMTV Env. A partial alignment of the viral protein Env is shown. The alignment includes all available rodent MMTV and MMTV-like virus sequences, as described in more detail in the manuscript, including the endogenous retrovirus found in the Peromyscus maniculatus genome. The TfR1 binding determinants of MMTV Env have not fully been mapped, but a TfR1-binding motif has been described [69] and is shown here in yellow. Changes from the MMTV sequence in this region are shown in bold. Viruses and endogenous retroviruses (ERVs) isolated from each of the three species encode different residues in this motif, but the functional significance of this is unknown. (PDF) [file pbio.1001571.s002.pdf]

Demogines et al

Figure S2

```
                                                          Putative TfR1
                                                          Binding Motif
          MMTV isolate C3H       NTDPIRVLTNQTIYLGGSPDFHGFRNMSGNVHFEGKSDTLPICFSFSFSTPTGCFQVDKQ
          MMTV isolate AF033807  STDPIRVLTNQTMYLGGSPDFHGFRNMSGNVHFEGKSDTLPICLSFSFSTPTGCFQVDKQ
house     MMTV isolate HeJ       STDPIRVLTNQTMYLGGSPGFHGFRNMSGNVHFEGKSDTLPICFSFSFSTPTGCFQVDKQ
mouse     MMTV isolate D16249    STDPIRVLTNQTMYLGGSPDFHGFRNMSGNVHFEGKSDTLPICLSFSFSTPTGCFQVDKQ
          Mmusc ERV ch4          STDPIRVLTNQTMYLGGSPDFHGFRNMSGNVHFEEKSDTLPICFSFSFSTPMGCFQVDKQ
          Mmusc ERV ch6          STDPIRVLTNQTMYLGGSPDFHGFRNMSGNVHFEGKSDTLPICFSFSFSTPTGCFQVDKQ
          Mmusc ERV ch12         STDPIRVLTNQTMYLGGSPDFHGFRNMSGNVHFEGKSDTLPICLSFSFSTPTGCFQVDKQ

          Rat ERV ch13           VSDHIKVLTNQTLVIGGSPDFHLLKNSSGYVDFEGKSDSLPICFSFSPSVPVGCFQVGKR
          Rat ERV ch19           VSDHIKVLTNQTLVIGGSPDFHLLKNSSGYVDFEGKSDSLPICFSFSPSVPVGCFQVGKR
rat       Rat ERV ch1            VSDHIKVLTNQTLVIGGSPDFHLLKNSSGYVDFEGKSDSLPICFSFSPSVPVGCFQVGKR
          Rat ERV ch15           VSDHIKVLTNQTLVIGGSPDFHLLKNSSGYVDFEGKSDSLPICFSFSPSVPVGCFQVGKG
          Rat ERV ch4            VSDHIKVLTNQTLVIGGSPDFHLLKNSSGYVDFEGKSDSLPICFSFSPSVPVGCFQVGKG
                                   * * ******   **** **   * ** * ** *** **** *** * * ***** *

          P.man ERV              DREPIKVLTNDSVRLGGAQDSDARSSSSSLVNFEGRADSLSICLTLQGKVPYGCFPTSYR
                                     * ****     **          *  * **   * * **       * ***
```

Partial ENV alignment of MMTV-like viruses and endogenous retroviruses (ERVs) of rodents
