# Supplementary material for: Dual Host-Virus Arms Races Shape an Essential Housekeeping Protein
Source: PLoS Biol. 2013 May 28;11(5):e1001571. doi: 10.1371/journal.pbio.1001571 (PMC3665890; doi:10.1371/journal.pbio.1001571)
Supplement: Table S1 — PAML analysis of rodent TFR1 sequences. This table summarizes the codon-based analysis of dN/dS performed on rodent TFR1 sequences. (PDF) [file pbio.1001571.s003.pdf]

Table S1. PAML analysis of rodent *TFR1* sequences.

TfR1[a] dataset	ω_0[b]	codon freq.[c]	*M1a-M2a* 2δ[d]	*M1a-M2a* p-value	*M7-M8* 2δ[d]	*M7-M8* p-value	*M8a-M8* 2δ[d]	*M8a-M8* p-value	tree length[e]	dN/dS (%)[f]	Residues with dN/dS>1 [g] * p>0.95
rodents	0.4	f61	15.8	p<0.0004	20.9	p<0.0001	15.6	p<0.0001	0.94	4.0 (2.9%)	109R(0.89), 205K, 209L*, 215N*, 296S, 569T(0.89), 575E*
	0.4	f3x4	14.9	p<0.0006	19.6	p<0.0001	14.3	p<0.0002	0.93	3.3 (4.0%)	109R, 205K, 209L*, 215N*, 296S, 569T, 575E*
	1.6	f61	15.8	p<0.0004	20.9	p<0.0001	15.6	p<0.0001	0.94	4.0 (2.9%)	109R(0.89), 205K, 209L*, 215N*, 296S, 569T(0.89), 575E*
	1.6	f3x4	14.9	p<0.0006	19.6	p<0.0001	14.3	p<0.0002	0.93	3.3 (4.0%)	109R, 205K, 209L*, 215N*, 296S, 569T, 575E*

a Dataset consisted of the aligned rodent sequences *Neacomys spinosus* (FJ154604), *Calomys callosus* (EU164540), *Cricetulus griseus* (L19142), *Mus musculus* (NM_011638), *Zygodontomys brevicauda* (EU340259) , *Calomys musculinus* (EU164541), *Rattus norvegicus* (NM_022712).

b Initial seed value for ω (dN/dS) used

c Model of codon frequency

d Twice the difference in the natural logs of the likelihoods (ΔlnL x 2) of the two models being compared. This value is used in a likelihood ratio test along with the degrees of freedom. In all cases (M1a-M2a), (M7-M8), (M8a-M8), a model that allows positive selection is compared to a null model. The p-value indicates the confidence with which the null model can be rejected.

e The tree length is the number of substitutions per site along all branches in the phylogeny. It is calculated as the sum of the branch lengths, and is a representation of total diversity in the dataset

f dN/dS value of the class of codons evolving under positive selection in M8, and the percent of codons falling in that class.

g Posterior probabilities of codons under positive selection in M8 were inferred using the Naive Empirical Bayes (NEB) algorithm. Residues listed correspond to codons assigned to the dN/dS>1 class with posterior probability P > 0.90 (by NEB), or with the indicated posterior probability. The criteria for inclusion in our functional study was that the site must be supported at a level P > 90% in one or more of these model permutations. Coordinates and animo acids listed correspond to the human protein, although the human sequence was not used in this analysis. Residue 109 lies outside of the TfR1 ectodomain structure and therefore was not analyzed further in the present study.
