# Supplementary material for: Dual Host-Virus Arms Races Shape an Essential Housekeeping Protein
Source: PLoS Biol. 2013 May 28;11(5):e1001571. doi: 10.1371/journal.pbio.1001571 (PMC3665890; doi:10.1371/journal.pbio.1001571)
Supplement: Table S2 — PAML analysis of Machupo virus gp1 sequences. This table summarizes the codon-based analysis of dN/dS performed on Machupo virus gp1 sequences. (PDF) [file pbio.1001571.s004.pdf]

Table S2. PAML analysis of Machupo *gp1* sequences.

| dataset[a] | $\omega_0$[b] | codon freq.[c] | *M1a-M2a* $2\delta$[d] | *M1a-M2a* p-value | *M7-M8* $2\delta$[d] | *M7-M8* p-value | *M8a-M8* $2\delta$[d] | *M8a-M8* p-value | tree length[e] | dN/dS (%)[f] | Residues with dN/dS>1[g] *p>0.95 ** p>0.99 |
|---|---|---|---|---|---|---|---|---|---|---|---|
| | 0.4 | f61 | 16.2 | p=0.0003 | 23.2 | p<0.0001 | 21.4 | p<0.0001 | 1.2 | 2.9 (6.7%) | 114** 115** 119** 122* 145** 170** 185** 206** 212** 230** 231** |
| | 0.4 | f3x4 | 8.1 | p=0.0176 | 11.7 | p=0.0029 | 10.0 | p=0.0015 | 1.2 | 2.1 (7.8%) | 114** 115** 119* 122** 145** 170** 185** 206* 212** 230** 231** |
| | 1.6 | f61 | 16.2 | p=0.0003 | 23.2 | p<0.0001 | 21.4 | p<0.0001 | 1.2 | 2.9 (6.7%) | 114** 115** 119** 122* 145** 170** 185** 206** 212** 230** 231** |
| | 1.6 | f3x4 | 8.1 | p=0.0176 | 11.7 | p=0.0029 | 10.0 | p=0.0015 | 1.2 | 2.1 (7.8%) | 114** 115** 119* 122** 145** 170** 185** 206* 212** 230** 231** |

a Dataset consisted of the aligned Machupo virus glycoprotein 1 (*gp1*) gene sequences from strains MARU223671 (AY571929.1), MARU221600 (AY571922.1), MARU223455 (AY571921.1), 9301012 (AY571919.1), 9301013 (AY571918.1), Chicava (AY571920.1), MARU258667 (AY571930.1), Mallele (AY619645.1), FSB 2040 (FJ696411.1), FSB 2334 (FJ696414.1), 9430071 (AY571912.1), MARU250720 (AY571917.1), MARU222688 (AY571916.1)

b Initial seed value for $\omega$ (dN/dS) used in the maximum likelihood simulation

c Model of codon frequency

d Twice the difference in the natural logs of the likelihoods ($\Delta$lnL x 2) of the two models being compared. This value is used in a likelihood ratio test along with the degrees of freedom. In all cases (M1a-M2a), (M7-M8), (M8a-M8), a model that allows positive selection is compared to a null model. The p-value indicates the confidence with which the null model can be rejected.

e The tree length is the number of substitutions per site along all branches in the phylogeny. It is calculated as the sum of the branch lengths, and is a representation of total diversity in the dataset

f dN/dS value of the dN/dS>1 class of codons in M8, and the percent of codons falling in that class.

g Posterior probabilities of codons under positive selection in M8 were inferred using the Naive Empirical Bayes (NEB) algorithm. Codons listed were assigned to the dN/dS > 1 class in M8 with P > 0.90 by NEB.
